# Exploring the Psychosocial Influences on Hepatitis B and Liver Cancer Disparities

**DOI:** 10.1007/s40615-025-02580-w

**Published:** 2025-08-25

**Authors:** Suzanne J. Block, Beatrice Zovich, Fiona Borondy-Jenkins, Thomas Chen, Kate Moraras, Bright Ansah, Chari Cohen

**Affiliations:** 1 Johns Hopkins University Bloomberg School of Public Health, Baltimore, MD USA; 2 Hepatitis B Foundation, 3805 Old Easton Road, Doylestown, PA 18902 USA; 3 The Mount Sinai Hospital, 1468 Madison Ave, New York, NY 10029 USA

**Keywords:** Hepatitis B, Liver cancer, Psychosocial factors, Immigrant health, Health disparities

## Abstract

**Background:**

Diverse communities from Asia, the Pacific Islands, the Caribbean, and Africa are disproportionately impacted by chronic hepatitis B. Low screening rates and limited engagement with disease management contribute to higher rates of liver cancer. This qualitative study aimed to explore the psychosocial factors influencing health behaviors related to hepatitis B and liver cancer, to inform the development of a public health communication campaign tailored to highly impacted communities.

**Methods:**

In collaboration with community partners, individuals identifying with each community were purposively selected to participate in this study. Fifteen focus groups, ranging in size from 7 to 12 participants, and two key informant interviews were conducted with individuals from Asian and Pacific Islander, Caribbean, and African communities in the United States (total *n* = 101). The Health Belief Model and Social Ecological Model guided initial codebook development, with inductive approaches capturing emergent themes identified during analysis.

**Results:**

Participants described incomplete knowledge of hepatitis B transmission and its link to liver cancer within their communities. These influenced health beliefs and behaviors surrounding screening and care, shaping feelings of self-efficacy and uncertainty regarding prevention and management. Misperceptions about disease risk and management were reproduced within interpersonal relationships, with many describing stigma and social isolation. Family support and culturally embedded social norms were influential in healthcare decisions and seeking.

**Conclusion:**

This study demonstrates the multi-level influences contributing to low rates of screening and care engagement across impacted communities, highlighting key differences and similarities. These findings reinforce the importance of culturally tailored education that reflects the diversity of communities affected by hepatitis B.

**Supplementary Information:**

The online version contains supplementary material available at https://doi.org/10.1007/s40615-025-02580-w.

## Introduction

Hepatitis B poses a significant public health threat as the most common cause of liver disease worldwide and a leading risk factor for hepatocellular carcinoma, or primary liver cancer [1, 2]. There are currently up to 2.4 million people living with chronic hepatitis B (PLWHB) in the United States [1, 2], with a disproportionate burden falling upon foreign-born individuals [3–5]. An estimated 60% of PLWHB in the U.S. emigrated from Asia, 19% from the Americas (predominantly from the Caribbean), and 15% from Africa [6]. These individuals also bear an increased risk of developing liver cancer [7–9]. Among foreign-born Asian and Pacific Islander (API) and African PLWHB in the U.S., the risk of developing liver cancer is 11 to 17 times higher than that of their white counterparts [10].

Hepatitis B is a viral infection transmitted through contact with blood or certain bodily fluids that invades the liver, causing either acute or chronic infections [11]. Mother-to-child transmission due to blood exchange during childbirth is the most common route of transmission for PLWHB born in countries with intermediate or high endemicity of hepatitis B [3]. A safe and effective vaccine exists to protect against hepatitis B, and for PLWHB, an earlier diagnosis, routine clinical monitoring, and appropriate antiviral treatment can stop or slow the progression to severe liver disease and liver cancer [11].

In recent years, there have been efforts to simplify screening and treatment guidelines in the U.S. [12], and some interventions aimed at promoting screening [13, 14]. These include using a specialized mobile application and social media to provide educational videos and materials to increase hepatitis B screening [13, 14]. Still, significant gaps in healthcare access and long-term care engagement persist [15]. Less than half of PLWHB in the U.S. are aware of their diagnosis, and for those who are aware, 28% receive treatment [16].

Such gaps are reflective of the barriers faced by heavily impacted foreign-born communities in the U.S. that are multi-level, spanning individual knowledge and beliefs, interpersonal dynamics, and community and social contexts. There is a lack of knowledge and awareness about hepatitis B and its risk of causing liver cancer, which contributes to consistently low vaccination and screening rates [17–19]. Consequently, myths and false perceptions about how or why someone has hepatitis B or liver cancer continue to shape negative attitudes and beliefs within communities [17, 20, 21]. This culminates in experiences of stigma, social isolation, and other negative experiences that exacerbate poor healthcare behaviors.

These multi-level psychosocial barriers contributing to the disproportionate burden of hepatitis B and liver cancer among foreign-born PLWHB can be understood through a socioecological lens. The socioecological model (SEM) asserts that no single factor is responsible for health behaviors and outcomes. Rather, varied factors across multiple levels interact to influence health behaviors and outcomes [22]. The Health Belief Model (HBM) helps to explain further how individuals navigate health behaviors within a socioecological framework. HBM suggests that an individual’s perceived threat of an illness, self-efficacy, or belief in their ability to perform the recommended health behavior, and their belief in the effectiveness of the behavior collectively influence its uptake [23]. HBM also examines the perceived benefits of and barriers to performing a desired health behavior. With this understanding, psychosocial factors that are perceived as barriers or benefits across various socioecological levels influence behaviors.

Insufficient literature examines the varied multi-level influences among specific ethnic groups heavily impacted by hepatitis B and liver cancer. This is a critical step for public health efforts seeking to increase screening, vaccination, and care to reduce the impact of these diseases among diverse communities effectively. The present study sought to address this gap by examining the multi-level psychosocial influences across underlying health perceptions and behaviors related to hepatitis B and liver cancer in higher-risk API, African, and Caribbean communities in the U.S. This will provide a nuanced understanding of the various influences, highlighting differences and similarities that will allow for culturally tailored education and interventions for each community.

## Methods and Materials

### Study Context and Design

#### Learn the Link Campaign

The data collected for this study were part of a larger project that informed the development and dissemination of a culturally and linguistically appropriate, community-focused awareness and education campaign. This campaign, later named Learn the Link [24], aimed to improve knowledge about the link between hepatitis B and liver cancer, reduce liver-cancer-related myths and misconceptions, and promote hepatitis B and liver cancer screening, and early detection behaviors among API, African, and Caribbean immigrant communities in the U.S. Communication materials were tailored to each community based on findings from this study. To date, there are materials geared towards twelve different communities, and they can be printed or shared on social media and other digital channels. Additional multimedia materials for each community are under development. Details about the design of the communication channels and messages preferred by each community have been described elsewhere [25, 26].

#### Community Collaborations

At the start of the project, an advisory committee was assembled with members from the API, African, and Caribbean communities. The committee comprised public health professionals, healthcare providers, people with lived experience of hepatitis B, and representatives of community-based organizations from around the country. These individuals were recruited through an extensive and highly developed network of advocates in the hepatitis B and liver cancer fields, which the research team had cultivated over many years.

Committee members had extensive knowledge of hepatitis B and/or liver cancer and represented communities highly impacted by these diseases. The committee’s purpose was to assist in assembling representative focus groups and guide the creation of research materials. This committee contributed to the research process by advising on the research methods, offering guidance on the particular communities with which to hold focus groups, identifying community members to facilitate and participate in focus groups, ensuring that the sample of participants was robust and diverse, and providing additional insights and perspectives into the challenges of addressing hepatitis B and liver cancer in these highly impacted communities. A focus group guide was developed in collaboration with the API and the African and Caribbean Advisory Committee to ensure cultural relevance and sensitivity.

### Study Sample and Recruitment

Fifteen focus groups were conducted throughout the U.S. from April 2021 through September 2021 using Zoom (one group was held in person in New York City and was audio-recorded for analysis). Among these were two groups conducted with members of the project’s advisory committee—one composed of API members and the other composed of African and Caribbean members. Two key informant interviews were conducted with advisory committee members who were unable to attend their respective advisory committee focus groups. The remaining thirteen groups were conducted directly with members of several different specific communities highly impacted by hepatitis B and liver cancer. These focus groups, which ranged in size from 7 to 12 participants [27], were recruited by local community leaders. A purposive sampling strategy was used to ensure that each priority community was represented. Focus groups were based on membership in the immigrant community. The following communities were represented: Micronesian, Chinese (Mandarin-speaking and Cantonese-speaking), Hmong, Nigerian, Ghanaian, Vietnamese, Korean, Somali, Ethiopian, Filipino, Haitian, and Francophone West African. Inclusion criteria for participation included identifying as a member of API, Caribbean, or African immigrant communities and being over the age of 18. Participants did not have to be diagnosed with hepatitis B, although in some cases, participants disclosed this status during focus group discussions, depending on their comfort level.

Discussions with advisory committee members who were conducting the focus groups informed the decision on broad inclusion criteria and oversampling. As this was the first time the research team was engaging with some of these communities, oversampling ensured a sufficient number of participants. This decision, along with broad inclusion criteria, enabled a range of perspectives and experiences to be represented within each community. This sampling strategy added richness to discussions and helped ensure saturation of findings within and across focus groups.

### Data Collection

Focus group members resided in cities around the United States, including Sacramento, Minneapolis, Seattle, Honolulu, San Diego, Los Angeles, New York City, Philadelphia, Miami, Washington D.C., Chicago, and Boston. Sociodemographic information was collected before each focus group, using a digital survey. This included age, residential setting (urban, rural, or suburban), place of birth (inside or outside the U.S.), and the length of time they had been in the U.S. The participant’s level of education was not asked, based on advisory committee recommendations that this was a culturally sensitive question. To further ensure cultural sensitivity, focus groups were conducted by trained community leaders. The focus group leader determined the language used in each focus group and provided cultural context for questions, when needed. For the groups for which translation was desired, these guides were translated into the appropriate languages (Cantonese, Haitian Creole, French, Korean, Vietnamese, and Marshallese) by a certified translation company (Elite TransLingo [28]) and reviewed by bilingual advisory committee members prior to use. The guides helped ensure consistent data collection, allowing for accurate comparisons during the analysis phase.

All focus groups were 60–90 min in length, and upon completion, participants were compensated with a gift card. The focus groups were recorded, and after each session, the audio recordings were transcribed and translated into English, if needed. Transcriptions and translations were completed professionally by DataGain Services [29].

### Data Analysis

Using NVivo 20 software [30], thematic coding and analysis were conducted to identify and explore the psychosocial influences that promote or prevent health-protective behaviors surrounding hepatitis B and liver cancer among heavily impacted communities. The research team developed a codebook a priori based on the focus group discussion guide and SEM [22] and HBM [23]. Constructs from the HBM were incorporated into the SEM’s individual, interpersonal, and community levels. Descriptive coding was used to summarize text segments. It included codes such as knowledge and awareness of hepatitis B and liver cancer, perceived disease risk, control over disease (self-efficacy), and stigma. Descriptive codes provided the basis for an iterative and interpretative coding process that reflected emerging themes and their nuance. The final overarching themes were categorized by SEM level and include relationships between various components of the health belief model: individual health beliefs such as knowledge about hepatitis B transmission shaping perceived threat of illness; varied knowledge of the hepatitis B and liver cancer relationship complicating perceived threat; perceived self-efficacy and sense of control over hepatitis B and liver cancer affecting prevention and treatment uptake; individual and interpersonal experiences of stigma posing barriers to screening and care; interpersonal interactions informing healthcare decisions; and community-level norms that influence healthcare-seeking (See Table 1 Supplemental File for a table of illustrative quotes for each theme and subtheme).

Throughout this process, the research team held weekly debriefing sessions to discuss the findings, emerging themes, and whether the codebook was capturing the data. To further enforce the rigor of the findings, each transcript was assigned a primary and secondary coder. Transcripts were coded independently. To ensure inter-coder reliability, primary and secondary coders met and discussed each transcript to resolve discrepancies and to make final decisions regarding textual analysis. The final kappa coefficient was 0.78, indicating high inter-coder reliability.

## Results

### Participant Characteristics

Table 1 describes the demographic characteristics of the focus group participants (*n* = 101). The average age of the participants was 52 years, and most lived in urban areas (83%). Moreover, 91% of all participants were born outside the U.S., although most of those in the Hmong focus group were born in the U.S. (83%). The average length of time in the U.S. among all focus group participants was 25 years. For the Francophone West African focus group, the average length of time in the U.S. was 12 years.

**Table 1:** Demographic characteristics of focus group participants

			Residence *n* (%)		Born in U.S *n* (%)		
	Overall n (%)	Mean age in years^1^	Urban	Suburban	No	Yes	Mean length of time in U.S. in years
	101 (100)	52	84 (83)	16 (17)	92 (91)	8 (9)	25
Community Group							
Chinese (Cantonese)	6 (6)	67	6 (100)	–	6 (100)	–	39
Chinese (Mandarin)	6 (6)	37	3 (50)	3 (50)	5 (83)	1 (17)	22
Filipinx	8 (8)	65	8 (100)	–	8 (100)	–	40
Hmong	6 (6)	36	3 (50)	3 (50)	1 (17)	5 (83)	23
Korean	9 (9)	55	9 (100)	–	9 (100)	–	19
Micronesian	5 (5)	45	5 (100)	–	5 (100)	–	24
Vietnamese	10 (10)	58	10 (100)	–	10 (100)	–	26
Ethiopian	6 (6)	54	3 (50)	3 (50)	6 (100)	–	32
Nigerian^2^	9 (9)	63	9 (100)	-	9 (100)	–	35
Nigerian and Ghanian^2^	6 (6)	53	2 (33)	4 (67)	6 (100)	–	18
West African^2^ (Francophone)	13 (13)	49	13 (100)	–	13 (100)	–	12
Somali	8 (8)	42	8 (100)	–	7 (88)	1 (13)	20
Haitian	9 (9)	58	9 (100)	–	9 (100)	-	20

^1^Participant age was not asked directly in any focus group. Instead, age was determined by accounting for age upon arrival in the United States and length of time spent in the United States

^2^Three distinct focus groups were held with West African participants: Nigerian participants only, Nigerian and Ghanian participants only, and French-speaking participants from multiple West African countries

### Psychosocial Influences

**Fig. 1:**
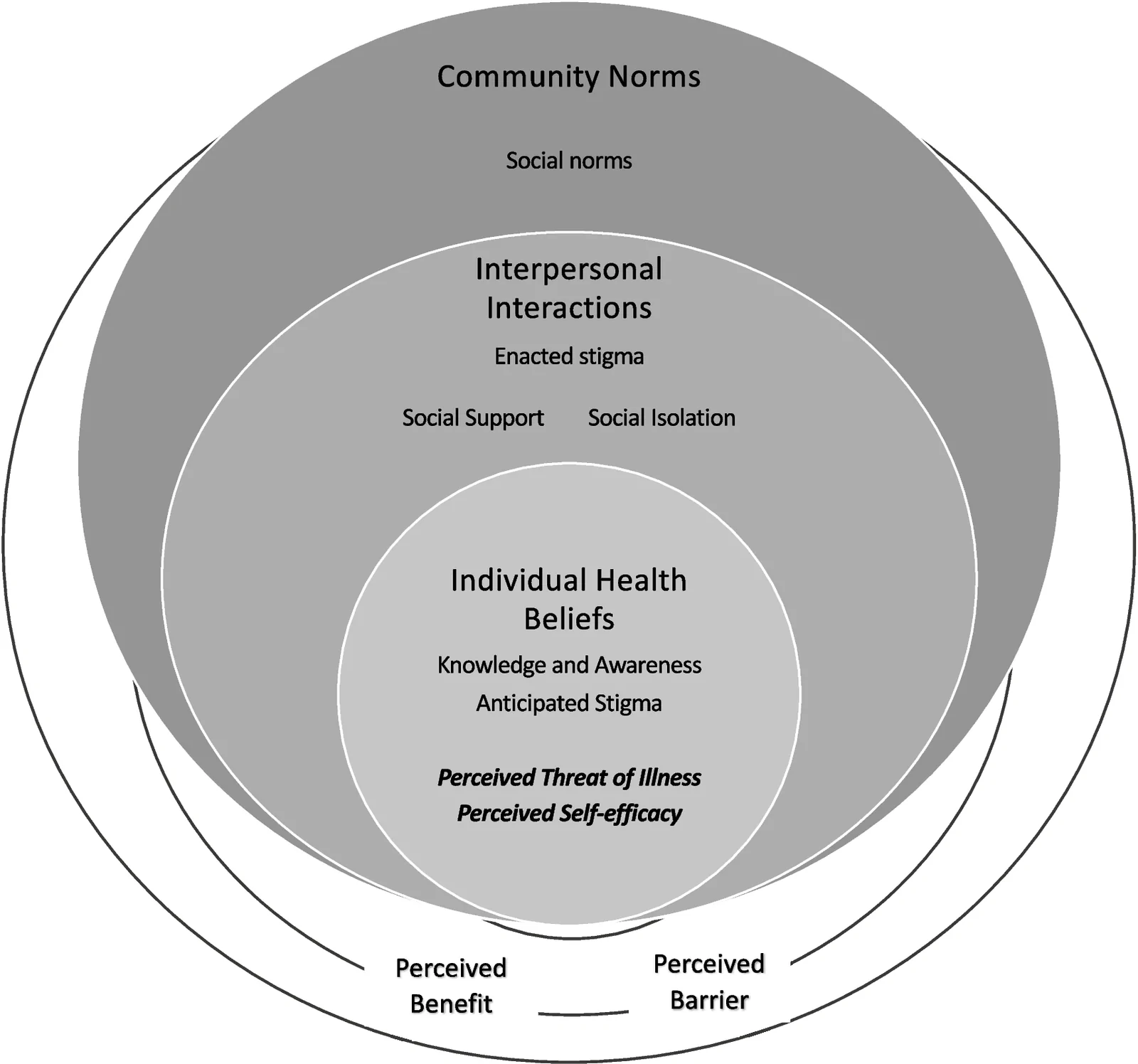
The psychosocial influences of hepatitis B and liver cancer health behaviors

Figure 1 is a visual representation of the identified psychosocial influences and their relationships. The study findings are organized based on their location within the SEM and how they may inform health behaviors related to hepatitis B and liver cancer. There are individual health beliefs shaped by knowledge, awareness, and stigma, interpersonal interactions that reproduce such beliefs and where individuals may experience stigma and social support or isolation, and community-level norms embedded in one’s culture. Altogether, these interact within and between levels, serving as a barrier or benefit to screening and overall care engagement. See Table 1 Supplemental File for a table of illustrative quotes for each theme and subtheme.

#### Individual Health Beliefs

##### Knowledge of Hepatitis B Transmission Shapes Perceived Threat of Illness

Focus group (FG) participants generally understood that hepatitis B is a transmissible disease. However, if participants did not have a medical background or experience through a personal diagnosis or from a family member, their understanding of the full spectrum of specific transmission routes varied. This understanding shaped the perceived risk of contracting hepatitis B. Most FGs described mother-to-child transmission, sexual transmission, and how it is passed through blood and bodily fluids. A Hmong participant explained how hepatitis B was understood to be something their community lives with, as it is “passed on from our parents down to the kids.” A Haitian participant explained: “It is something that passes through your blood, and it is sexual, so people should be very careful.”

The knowledge that hepatitis B transmission could occur through contact with blood, semen, and other bodily fluids gave rise to uncertainty among some participants and was conflated with unrelated modes of transmission. A Mandarin-speaking participant described how people in their community believe it is something you can “catch,” and several FGs shared stories of not sharing utensils or food within households. One participant from the Nigerian and Ghanian FG discussed the inevitability of hepatitis B infection because of this misunderstanding: “if you’re living with anybody that has it… I mean, sharing everything with them, like a spoon, whatever, I think you can have it if you don’t have it before [sic]. So that’s the only thing, and I don’t think anybody has control.”

##### Varied Knowledge of the Hepatitis B-Liver Cancer Relationship Complicates Understanding of Perceived Threat of Illness

The knowledge that hepatitis B can cause liver cancer varied within and among community FGs. Some recognized that they had previously not distinguished between the two to understand the causal relationship: “My first thought before was that the two are the same, but I just learned today that it’s two totally different illnesses that someone has” (Micronesian FG Participant).

FGs, including those from the Somali and Francophone West African groups, discussed the difficulty in making this distinction due to hepatitis B’s silent nature and disease progression. A Francophone West African participant described how hepatitis B is an “insidious disease…and that in the end, it can lead to cancer.” Other FG participants attributed their knowledge of the causal relationship to their professional background in the medical field or personal experience with family members. One Hmong participant shared: “I learned it [sic] for the first time when my mom was diagnosed with it, and I didn’t realize it. I mean, I knew that hepatitis B affected the liver, but I didn’t realize that it could cause cirrhosis and liver cancer if not treated.”

While there was variability in personal and professional experiences that informed the understanding of the causal relationship between hepatitis B and liver cancer, the association of liver cancer with alcohol consumption was pronounced across FGs. A participant from Ethiopia explained the pervasive nature of this association and how this belief may eclipse perceptions of other causes of liver cancer, such as hepatitis B: “When I was in Ethiopia, I myself used to think that anyone who is ill with liver disease is a drunk. To be honest, I used to think it is because the person is alcohol abuse [sic], and I never thought liver [disease] had another cause.”

##### Perceived Self-Efficacy and Sense of Control over Hepatitis B and Liver Cancer Diagnosis Affected Prevention and Treatment Behavior

The perceived self-efficacy to control a diagnosis and take appropriate action was reflective of the knowledge and awareness that participants held regarding how hepatitis B is transmitted and its connection to liver cancer. FG participants described hepatitis B testing as an effective way to take control and proactively manage their risk. A Chinese-Mandarin FG participant also differentiated their ability to control hepatitis B as compared to liver cancer:

For hep B, no, because I contracted that when I was little, but for liver cancer, absolutely. For managing hep B successfully and diligently decreases my risk of liver cancer; so I think that’s where the control is exerted. And I also have control on preventing my loved ones [from getting it].

Those in the Vietnamese and Chinese-Mandarin FGs made the assertion that they could manage their disease to prevent liver cancer. It was also commonly discussed across FGs that liver cancer prevention or progression could be controlled through lifestyle choices. A participant from the Filipino FG mentioned, “I think we have control if we manage our lifestyle” and another participant from the Cantonese**-**speaking FG said, “If you have enough sleep and nutrients, then you can stop it from showing effect.”

Other community FGs discussed prevention methods in the context of faith. An individual in the Korean FG said, “Your faith life, along with efforts to prevent hepatitis, can have a positive impact.” A Somali participant provided reasoning for their health behaviors related to faith: **“**When I think of faith, I think of the plan of God, right? So, I think…whatever is destined for us will come. However, I do feel like we do have a level of control of whether we get the virus.”

#### Individual and Interpersonal Experiences of Stigma Surrounding Hepatitis B and Liver Cancer Often Pose Barriers to Screening and Care

Misperceptions about hepatitis B and liver cancer contributed to disease-related stigma experiences and were commonly referenced as barriers to screening and care. One Cantonese-speaking participant detailed feelings of anticipated or perceived stigmatizing attitudes that others may hold against someone with hepatitis B: “People just assume that your living conditions are bad, or you grew up in a family that doesn’t care about hygiene.” A participant from the Micronesian FG shared a potential scenario of enacted stigma: “When they find out that they have hepatitis B, they may treat them differently, or worse, just outright try to avoid them.” A member of the African and Caribbean Advisory Committee discussed how hepatitis B is perceived solely as a sexually transmitted disease in their communities, informing stigmatizing attitudes and experiences of enacted stigma:

So anything that could even remotely be transmitted through sexual intercourse is always seen as the source of stigma. It is seen to refrain from coming out just because it could be viewed as a matter of being acquired through being promiscuous or unfaithful. And on the other side, it also provides stigma by others who may see them as such [sic]. And that’s always been a hindrance in the process to help people.

Lastly, a Ghanaian key informant interviewee highlighted the difficulty in changing these stigmatizing perceptions: “There are some people who still, after all the education you can give them… still have lingering traditional myths and misconceptions…it’s just so powerful.”

#### Interpersonal Interactions Informing Healthcare Decisions for Hepatitis B and Liver Cancer

##### Fear of Social Isolation Deters Hepatitis B Screening

The social consequences of being diagnosed with hepatitis B instilled fear and avoidance of care-seeking, including screening, among many communities. Consequences might stem from disease-related stigma and result in rejection and isolation from social networks. A Micronesian FG participant said, “We share almost everything. Our culture is about community …when someone is treated with these diseases, there is a sense of you being left out and being abandoned.” A Hmong FG participant also shared:

There’s a lot of people out there just fear [sic] of knowing that you might have hepatitis B. You don’t want to accept the truth. For me, for example, if I were ever sick, I just feel I don’t want to know what’s wrong with me, like, “What if I do have something? And I do not know how to tell my family, or I don’t want my family to worry.” So, for me, it’s just fear of the truth.

Different FGs, including those of the Cantonese-speaking, Micronesian, West African, and Haitian communities, also discussed the anticipated consequences of being diagnosed with hepatitis B. A Francophone West African participant said, “I think it’s a fear of being rejected because, in the community, there are certain diseases, where if others find out, knowing that it’s contagious…they’re a bit restrained.”

##### Social Support Encourages Healthcare-Seeking Decisions

Some participants described the role of social support in their healthcare decisions, including when to disclose and when to seek care. The API Advisory Committee explained how some in their communities, particularly Hmong, make decisions that are guided by family and faith: “If they do hold to [sic] a lot of those old religious beliefs, then perhaps instead of going to get tested or get screened, what they’re going to do first is maybe call their family, call the shaman and do what they need to do first.” A key informant shared an example characterizing how their Ghanian community members also rely on family members when deciding whether to be screened for hepatitis B: “They might say, ‘I will only do it if my family member also does it’…So, if they have someone who really understands the danger and is there to support them and is not going to say, ‘Well, you are on your own kind of.’”

Some, such as those in the Nigerian and Vietnamese FGs, did not communicate hesitancy in seeking family support. One Vietnamese participant highlighted how sharing could increase the amount of support they receive and expand their care networks: “When we share to other people, they will take care of us more, ask more about us, that help our mind getting more positive.”

#### Community-Level Culturally Embedded Social Norms Influence Health Behaviors

##### Hiding Diagnoses as a Cultural Practice

Hiding a diagnosis was commonly cited as a health behavior rooted in cultural practices. An Ethiopian FG participant explained, “Any disease is hidden in our culture. You don’t speak about it openly, especially if it is cancer.” A participant from the Haitian FG echoed this sentiment: “As for us Haitians, we are always hiding our disease, like the disease that people are afraid of.” A participant from the Nigerian and Ghanaian FG added, “There is a cultural effect on that [sic], and because people are concerned about confidentiality, that tends to hold them back from even letting others know what kind of disease or what medical condition they are going through [sic].” A Micronesian participant further explained:

With regards to liver cancer, or any other disease, men and women in the Marshall Islands tend to keep it to themselves because they say they can bear it, they can grin and bear it and keep working, no matter what they feel, no matter the pain, the symptoms, until it becomes too late when they’re very sick, so this could be a cultural thing that becomes a problem.

##### Healthcare-Seeking Involves Navigating Western and Traditional Medicine Due to Culturally Embedded Social Norms

There was variation in healthcare-seeking preferences driven by culturally embedded norms within and between community FGs. Some key informants from the Western Pacific regions shared that many people in their communities utilize both traditional and Western medicine. Other FGs discussed community preferences for holistic or traditional healing methods that lay in tension with seeking or remaining engaged in care within Western medical settings. An Ethiopian participant explained that some believe that traditional medicine can cure hepatitis B. Hmong participants also discussed this tension between culture and medical preferences:

In terms of those that are still practicing shamanism… I think it’s preventing our community from seeking treatment, and also continuing treatment, not just for liver cancer and hepatitis, but other medical conditions, because without the continuation of treatment, you’re going to allow the disease to further progress.

A participant from the Nigerian and Ghanaian FG shared their perspective on the role of privacy in their preferences toward non-Western medicine:

When you have a conversation with the doctors, the word gets out on the street. So, a lot of people stay away from actually getting a diagnosis, proper diagnosis, timely diagnosis, so they end up going to the herbal doctors, where privacy is a little bit assured.

## Discussion

This study explored the psychosocial factors influencing hepatitis B and liver cancer health behaviors among highly impacted communities within the U.S. to inform a national communication campaign. This campaign, called Learn the Link [24], aimed to develop culturally appropriate and tailored messaging for each community. Applying the HBM elucidated how PLWHB and those who are potentially at risk navigate healthcare decision-making and engagement in or avoidance of hepatitis B and liver cancer health behaviors based on knowledge and beliefs. Contextualizing one’s navigation within their socioecological landscape acknowledges that multiple influences operate interdependently across different levels. In this study, narratives of personal responsibility for preventing hepatitis B and liver cancer emerged during FG discussions. This responsibility had to be navigated within the social and cultural context of their community.

Knowledge about hepatitis B transmission and its causal relationship to liver cancer created a foundation for participants’ health beliefs and behaviors towards screening and care engagement. The general recognition among FGs that hepatitis B can be transmitted from person to person through blood and certain bodily fluids led to most voicing some degree of self-efficacy in preventing hepatitis B. PLWHB in FGs expressed the importance of disease management in preventing liver cancer, and several groups mentioned that engaging in healthy lifestyle behaviors, such as eating a healthy diet, avoiding alcohol, and exercising, was an important part of self-management. These mentalities instill a sense of personal responsibility, leaving room for both feelings of control and self-efficacy, as well as uncertainty surrounding the prevention or management of hepatitis B. This uncertainty is fueled by the misperception that hepatitis B can be spread by sharing food and utensils, as expressed by the Nigerian and Ghanaian, Micronesian, Korean, Mandarin-speaking, and other FGs. Lee et al. [31] similarly illustrate such misperceptions about hepatitis B transmission among Korean Americans and how key aspects of transmission through blood and certain bodily fluids remain unclear, challenging appropriate and protective health behaviors.

The lack of understanding of the causal relationship between hepatitis B and liver cancer is also consistent with other studies [18, 21]. One study focused on hepatitis B and liver cancer perceptions among ethnically diverse Black communities in South Florida, describing misperceptions about hepatitis B disease progression to liver cancer and the weight placed on causes of liver cancer, such as alcohol use, rather than living with hepatitis B [21]. These findings, along with another published study specifically focused on hepatitis B knowledge as part of the larger Learn the Link project, demonstrate the continued importance of addressing specific knowledge gaps about hepatitis B [32].

These narratives of personal responsibility to prevent hepatitis B and liver cancer, and common misperceptions about disease risk and management, are produced within interpersonal relationships; therefore, reaching social networks. Hepatitis B stigma is commonly reported in the literature, with a diagnosis associated with risk-taking behaviors such as drug use and sexual promiscuity [33–36]. This study adds to the literature, with participants across all groups describing how having hepatitis B or liver cancer can lead to false assumptions about their personal choices and social isolation. These stigma experiences served as barriers to screening among those at risk and barriers to care-seeking for PLWHB. Alternatively, some groups, such as the Vietnamese and Ghanaian, emphasized the supportive role of family and their social network, and this support was found to have the power to facilitate positive health behaviors.

Family is well-documented as being highly valued among foreign-born API and African communities, serving an important role in healthcare-seeking, including engagement with preventative services for hepatitis B [37–39]. This support is intertwined with cultural norms, which FGs described as strongly influencing when and where care is sought. Care-seeking preferences are rooted in trust, with FGs explaining their trust in traditional medicine. However, a mix of Western and traditional medicine may be used, with some studies finding that Western healthcare is sought after seeing a traditional healer or in cases of emergencies or sudden onset of symptoms [17, 40].

Overall, this study provides foundational knowledge for the Learn the Link communication campaign [24]. The findings on interpersonal and mediated (print and social media) communication are discussed elsewhere, highlighting specific preferences for messaging in each community [25, 26]. These current findings that informed the campaign’s communication messages demonstrate the multi-level influences contributing to hepatitis B and liver cancer health disparities. These include misperceptions and misunderstandings that shape health beliefs, including incomplete knowledge about the causal relationship between hepatitis B and liver cancer, stigma, and cultural norms. These findings are supported by and expand upon existing literature, providing insight into how these influences vary across communities, and the distinctions when discussing hepatitis B compared to liver cancer. They also provide a framework for how public health practitioners can effectively intervene in culturally relevant ways, ensuring that all those impacted are reached.

To this end, a greater emphasis on hepatitis B’s connection to liver cancer and the importance of healthcare engagement, including with Western medicine, to prevent or manage hepatitis B and liver cancer is needed in public health communication. For instance, the value placed on personal control through maintaining a healthy lifestyle indicates the existence of self-efficacy in engaging in health-protective behaviors. Educational messages should leverage this self-efficacy and direct behavioral choices to include medical management for hepatitis B or the prevention of liver cancer as an empowering choice for the individual. This messaging should also emphasize the causal relationship between hepatitis B and liver cancer. This type of messaging should also be delivered in tandem with messages that combat stigma and promote social support to further empower communities to take action and prevent the progression of hepatitis B to liver cancer.

The relationship between hepatitis B and liver cancer has not always been as strongly emphasized in other educational communication campaigns to increase knowledge and awareness about hepatitis B [41–43]. However, there are examples of making this connection, such as a social media intervention focused on hepatitis B screening and liver cancer, that have demonstrated the importance of doing so [14]. Altogether, such efforts demonstrate the effectiveness of patient education on learning the link between hepatitis B and liver cancer and communication campaigns through different channels to increase knowledge, awareness, and protective health behaviors among diverse communities.

Lastly, it is necessary to remember that other higher-level influences, such as policy and healthcare systems, impact the psychosocial influences at the individual, interpersonal, and community levels, which were the focus of this study. These upstream influences play a vital role in health behaviors and will be investigated in future research from this larger study.

## Lessons and Limitations

This study has several limitations and lessons learned. The results presented in this study are not generalizable to the broader U.S. population but rather are situated within the context of foreign-born communities with increased risk of hepatitis B living in the U.S.

Therefore, the results are transferable only to other populations with similar characteristics. The decision to use broad inclusion criteria, which allowed anyone identifying as part of a cultural community to participate, also prevented the research team from accurately differentiating between FG participant experiences and perspectives based on being diagnosed with hepatitis B. Participants were able to self-disclose, but this was not required. It must also be noted that some group discussions may also have social desirability bias because of group dynamics or have been influenced by some participants being clinicians or providers with prior knowledge about hepatitis B and liver cancer. One focus group was subject to leading questions and prompts from its facilitator, which was identified by the study team and accounted for in the analysis.

Challenges with technology and translation may also have impacted this study. This study required technological skills to use Zoom, and a lack of comfort using web-based services may have prevented some from fully participating in focus group discussions. Additionally, while the study used professional transcription and translation for focus groups conducted in languages other than English, there is potential for missed jargon or incorrect translations of words or phrases. This study also found that the focus groups themselves were often a source of awareness and knowledge of hepatitis B for participants. In the future, a small educational session following each group or providing a fact sheet or other resource for participants would be beneficial.

## Conclusion and Next Steps

There is a paucity of studies that explore psychosocial factors influencing hepatitis B and liver cancer health outcomes among at-risk and culturally diverse communities. This study adds important information to the current body of knowledge regarding the differences in psychosocial influences between impacted communities and the differing perceptions surrounding hepatitis B and liver cancer and their connection. These findings provide a substantial foundation from which to draw for the creation of robust and thoughtful health communication campaigns that address these particular concerns and barriers to screening, vaccination, and care experienced by these diverse communities in a nuanced way.

Future campaigns can directly address themes such as self-efficacy, uncertainty around Western medicine, stigma, and cultural norms, in addition to presenting accurate information about hepatitis B, liver cancer, and the causal relationship between the two. Such efforts can work to close the very real and pronounced health disparities caused by hepatitis B and liver cancer in these communities. To be effective, health communication should be personal and reflect the specific psychosocial and cultural needs within communities. The results of this study can inform the development of future communication campaigns to help address gaps in screening, effective management of hepatitis B, and early detection of liver cancer to improve health outcomes in a meaningful and sustainable way.

## Supplementary Information

Below is the link to the electronic supplementary material.

Supplementary file 1 (DOCX 3.75 MB)

## Data Availability

The codebook and interview guide will be made available upon reasonable request by corresponding author.
