# Supplementary material for: Exploring the Psychosocial Influences on Hepatitis B and Liver Cancer Disparities
Source: J Racial Ethn Health Disparities. 2025 Aug 25;13(5):4296–306. doi: 10.1007/s40615-025-02580-w (PMC12424198; doi:10.1007/s40615-025-02580-w)
Supplement: Supplementary file 1 [file 40615_2025_2580_MOESM1_ESM.docx]

Exploring the Psychosocial Influences on Hepatitis B and Liver Cancer Disparities

**Suzanne J. Block,^1^*, Beatrice Zovich^2^, Fiona Borondy-Jenkins^2^, Thomas Chen^3^, Kate Moraras^2^, Bright Ansah^2^, Chari Cohen^2^**

^1^Johns Hopkins University Bloomberg School of Public Health, Baltimore, MD

^2^Hepatitis B Foundation, 3805 Old Easton Road, Doylestown PA 18902 USA

^3^The Mount Sinai Hospital, 1468 Madison Ave, New York, NY 10029

*Corresponding author(s). E-mail(s): Sblock8@jhmi.edu

**Supplemental File 1**

**Table 1. Focus Group Discussion Quotes Table**

| **Theme** | | **Illustrative Quotes** |
| --- | --- | --- |
| **Individual Health Beliefs** | | |
|  | Knowledge of Hepatitis B Transmission Shapes Perceived Threat of Illness. | “Hepatitis B I've learned it back in nursing school, you know…it's a virus that affects the liver, it can also be transmitted by a blood transfusion, needle sticks, it's also sexually transmitted.” – Filipino FG Participant  “[My niece] contracted the Hepatitis B. She's very young. So, through her is what I learned, how deadly the disease and how easily can be transmitted through various way, not only sexually but also through body fluids and other means” – Somali FG Participant  “it's typically in our community, Hmong communities, passed on from our parents down to the kids.” -Hmong FG Participant  “it is something that passes through your blood and it is sexual, so people should be very careful.” -Haitian FG Participant  “It's a disease of the liver, and it's mostly transferred through sex or through blood, through mother child.” – Nigerian FG Participant  “I know that it's from blood. Yeah. You can't get it from say, saliva, but a lot of the ways people contracted is just from blood, or when you're born, and if your parent is a carrier, or your mom is a carrier, then that's how, in your bloodstream you might get Hep-B or that's how the viruses is passed.” – Chinese-Mandarin FG Participant  “I am scared of dirty things, so my home is always clean…I sometimes get cranky because I am always cleaning things. One of my relatives passed away due to hepatitis. I always thought hygiene was the reason why people were infected.” – Chinese-Cantonese FG Participant  “if you're living with anybody that has it… I mean, sharing everything with them, like spoon whatever, I think you can have it if you don't have it before. So that’s the only thing and I don’t think anybody have control.” - Nigerian and Ghanian FG Participant  “I don't know much about Hep B, but I also know that it's greater risk in our Micronesian community, and I'm not sure if it's a communicable disease, but I do know very, very small that it does spread through saliva as well, which it will portray as a bigger problem, especially that we share so many things at home, when it comes with food, and drinks, and in Hawaii, we have many, many generational home and if someone got infected, it's easier to spread in that home.” – Micronesian FG Participant  “I have heard that Koreans are at a much higher risk of contracting hepatitis because they share a one dish food such as soup.” – Korean FG Participant |
|  | Varied Knowledge of the Hepatitis B-Liver Cancer Relationship Complicates Understanding of Perceived Threat of Illness. | “My first thought before is the two are the same, but I just learned today that it’s two totally different illnesses that someone has” -Micronesian FG Participant  “I think Hepatitis B is important, but I don’t know if Hepatitis B and Cirrhosis are different?” – Vietnamese FG Participant  “I do know that it is a silent, insidious disease, that it attacks the liver, and that in the end, it can lead to cancer…[someone I knew] had liver cancer, but didn't find out until the last minute. It's a disease that is extremely silent.” -Francophone West African FG Participant  “Well, what caught my attention as to when this disease degenerates and turns into cancer, is because someone might be asymptomatic. They don’t know and they have the disease in them for years but not aware of it. And when they go to do a screening test it might be too late because it has been turned into cirrhosis or cancer” – Haitian FG Participant  “it's tough to connect between Hepatitis B and the cancer itself.” – Somali FG Participant  “it was associated with drinking, I never heard that it was transmittable other than what I got from reading later on.” – Ethiopian FG Participant  “I learned it for the first time when my mom was diagnosed with it, and I didn't realize it. I mean, I knew that hepatitis B affected the liver, but I didn't realize that it could cause cirrhosis and liver cancer if not treated.” -Hmong FG Participant_  “Well, I know nothing about hepatitis B, I know that it is a big illness among our community; I don't know the connection between with hepatitis B and liver cancer, but I think what I know of liver cancer is that my father passed away when he was 68 years old.”- Micronesian FG Participant  “When I was in Ethiopia, I myself used to think that anyone who is ill with liver disease is a drunk. To be honest, I used to think it is because the person is alcohol abuse, and I never thought liver [disease] had another cause.”- Ethiopian FG Participant  “I've been told that drinking too much alcohol can cause liver cancer. And sometimes it is caused by stress, and I know that if the parents had a history of liver cancer at birth, it could also happen to their children.” - Korean FG Participant  “for us in Haiti we are more focused on liver cirrhosis as something someone can get because they drink a lot of alcohol.” – Haitian FG Participant  “All I know is that major source of liver disease is heavy drinking.” – Filipino FG Participant  “I feel like we talk about how alcohol plays a role and how Hepatitis B can - if an individual has it, it can really increase their chances of having liver cancer.” - Hmong FG Participant  “Most of this is from my own experience and not just from the people that drink alcohol a lot now, the consumption of alcohol will really create a terrible damage on the liver” - Nigerian FG Participant |
|  | Perceived Self-efficacy and Sense of Control over Hepatitis B and Liver Cancer Diagnosis Affects Prevention and Treatment Behavior. | “If you're informed, and sick people know the symptoms, when they have information, they can run to get tested more quickly.”- West African FG Participant  “Hepatitis B, everybody can get it. That is why the screening. Once in your lifetime you have to get a screening test. If they didn’t take the vaccine, they have to do the screening. the screening is the thing that would help us know if we get it or not.” - Haitian FG Participant  “I’m a Hepatitis B carrier, but it stays in control…I’m not at that stage [liver cancer], so I have no idea. I only have experience as a Hepatitis B carrier, and my family asked me to do vaccination, follow up doctor, do blood test, follow up every six months with doctor to see if there is any progression.” - Vietnamese FG Participant  “For hep B, no, because I contracted that when I was little, but for liver cancer, absolutely. For managing hep B successfully and diligently decreases my risk of liver cancer; so I think that's where the control is exerted. And I also have control on preventing my loved ones [from getting it].” - Chinese-Mandarin-speaking FG Participant  “As for me, if I know for a fact that I do not have hepatitis, then I think it is controllable. I can stop myself from getting liver cancer. Even if you have hepatitis, you still need to stop it from turning into cancer." – Chinese-Cantonese-speaking FG Participant  “We have to be clear with the people about how they can get [hepatitis B]. Because they also have to change their lifestyles.” - Haitian FG Participant  “I think we have control if we manage our lifestyle” - Filipino FG Participant  “In my opinion, it is also related to your diet. For example, for Asians, we like to drink. When you are already infected, if you don't care about your liver health, the situation can become worse much quickly. Therefore, resting and diet are both important. People from the last generation work too much. They are always lack of sleep and tired. It makes them more vulnerable to the virus.” – Chinese-Cantonese-speaking FG Participant  I think lifestyle change is also important, you know. What do you do not to get there, what are the day-to-day things people can do to stay healthy? - Ethiopian FG Participant  “With people who are at higher risk, such as those with hepatitis B, or chronic alcoholics usually; they need some sort of support system in order to maintain their healthier lifestyle to prevent them to reduce their risk as much as possible.” - Micronesian FG Participant  “Your faith life, along with efforts to prevent hepatitis, can have a positive impact.” - Korean FG Participant  “When I think of faith, I think of the plan of God, right? So, I think…whatever is destined for us will come. However, I do feel like we do have a level of control of whether we get the virus.” - Somali FG Participant  “So, religious beliefs can be a – is also a challenge because they said, "Oh, God loves us, and God understand that I am that very serious and committed Christian and I do my part at church and do all the service and follow the foundation and the beliefs of the Christian entity. So, I will survive. And so, I don't need to take care of myself on the medicine side for treatment, because God will touch me. He has the healing hand and power."... if I tell them, then they will put me as a bad example of a Christian guy. He is against the, you know, he against our belief.” - Micronesian Key Informant Interviewees |
| **Individual and Interpersonal Experiences of Stigma Surrounding Hepatitis B and Liver Cancer Often Pose Barriers to Screening and Care** | | |
|  | *No subtheme* | "People just assume that your living conditions are bad, or you grew up in a family that doesn't care about hygiene.”- Chinese-Cantonese-speaking FG Participant  “When they find out that they have hepatitis B, they may treat them differently, or worse, just outright try to avoid them.”- Micronesian FG Participant  “So anything that could even remotely be transmitted through sexual intercourse is always seen as the source of stigma. It is seen to refrain from coming out just because it could be viewed as a matter of being acquired through being promiscuous or unfaithful. And on the other side, it also provides stigma by others who may see them as such. And that's always been a hindrance in the process to help people.” - African and Caribbean Advisory Committee FG Participant  “There are some people who still, after all the education you can give them… still have lingering traditional myths and misconceptions…it’s just so powerful.” – Ghanaian Key Informant Interviewee  “It's associated with being promiscuous. And it's highly stigmatized disease. And they know about it, and if somebody has it, it's stigmatized.” – Somali FG Participant  “People just tend to shy away from people with disease because they think they too might get it.” - West African FG Participant |
| **Interpersonal Interactions Informing Healthcare Decisions for Hepatitis B and Liver Cancer** | | |
|  | Fear of Social Isolation Deters Hepatitis B Screening | “We share almost everything. Our culture is about community …when someone is treated with these diseases, there is a sense of you being left out and being abandoned.”- Micronesian FG Participant  “There's a lot of people out there just fear of knowing that you might have hepatitis B. You don't want to accept the truth. For me, for example, if I was ever sick, I just feel I don't want to know what's wrong with me, like, ‘What if I do have something? And I don't know how to tell my family, or I don't want my family to worry.’ So, for me, it's just fear of the truth.” – Hmong FG Participant  “I don’t know, I guess culturally there are many things and we are not that open like people of this country, we are private. So, when these kinds of things happen in our lives, we rather keep it to ourselves, even for close family as well. So, I think we have that kind of culture.” – Ethiopian FG Participant  “Hepatitis B is considered a sexually transmitted disease that it could be looked down upon. And I think that’s where the shame may come in, and lack of sharing to others.” - Hmong FG Participant  “You feel like you are isolated and labelled.” - Chinese-Cantonese-speaking FG Participant  “I think it's a fear of being rejected because, in the community, there are certain diseases, where if others find out, knowing that it's contagious…so they're a bit restrained.”- Francophone West African FG Participant  “People who [have] it are scared others will keep away from those having it.Thinking it will kill them by interacting with anyone who has it…I knew about a husband abusing his wife [who] is a disease carrier just because he knew about it.” – Nigerian FG Participant  “I believe all that they are scared of is being isolated. They are scared not to get isolated.”- Haitian FG Participant  “We may lose connection with the rest of the community because they will become alienating us. So, that is scary and they don't wanna do.” - Micronesian Key Informant Interviewees |
|  | Social Support Encourages Healthcare-Seeking Decisions. | “If they do hold to a lot of those old religious beliefs, then perhaps instead of going to get tested or get screen, what they're going to do first is maybe call their family, call the shaman and do what they need to do first.”- API Advisory Committee FG Participant  “They might say, 'I will only do it if my family member also does it…So, if they have someone who really understands the danger and is there to support them and is not going to say, “Well, you are on your own kind of.” - Ghanaian Key Informant Interviewee  “If you know you have hepatitis, then you need to be more proactive. If you know that some family members have it, then you need to pay more attention on this matter. No matter you were born with it, or get infected later on in life, you need to take care of yourself and try to prevent it from progressing into liver cancer.” - Chinese-Cantonese-speaking FG Participant  “I think some people might share just because they want to make someone aware that you should probably get tested, especially your family.” - Hmong FG Participant  “It’s better for me to let people know because you don’t know who’s going to help you. They might have knowledge about the diseases process and treatment. By letting them know they can give you an advice on how to go about it, plus your family member also can be supportive.” - Nigerian FG Participant  “When we share to other people, they will take care of us more, ask more about us, that help our mind getting more positive.” - Vietnamese FG Participant |
| **Community-level Culturally Embedded Social Norms Influence Health Behaviors** | | |
|  | Hiding Diagnoses as a Cultural Practice. | “Any disease is hidden in our culture. You don’t speak about it openly, especially if it is cancer.” - Ethiopian FG Participant  “As for us Haitians, we are always hiding our disease, like the disease that people are afraid of.” - Haitian FG Participant  "There is a cultural effect on that, and because people are concerned about confidentiality, that tends to hold them back from even letting others know what kind of disease or what medical condition they are going through.” - Nigerian and Ghanian FG Participant  “With regards to liver cancer, or any other disease, men and women in the Marshall Islands tend to keep it to themselves because they say they can bear it, they can grin and bear it and keep working, no matter what they feel, no matter the pain, the symptoms, until it becomes too late when they're very sick, so this could be a cultural thing that becomes a problem.” – Micronesian FG Participant  “If somebody has a liver issue, or they are about to die, or they are in serious health condition, they will keep it to themselves or to the inner circle to ensure that it's not shared among - the community doesn't know about it…if you are about to die or in a deadly situation, you keep it to yourself, you keep it to your family. At the end of the day, you hear Mr. X or Mrs. X passed away, and they don't want to share it.” - Somali FG Participant  “So, I know that not just for hepatitis B, but for a lot of diseases and illnesses…Africans just don’t like to talk about things that are bothering them.” - West African FG Participant  “Like for the Filipinos, when they learn something that they have like a cancer, they don’t like to share it or broadcast it. They want to keep it on themselves.” - Filipino FG Participant  “I think this is also related to our traditions. Asian s and Chinese don’t want others to know about their pain, sufferings, and diseases…They don’t really want to talk about things like cancers. I think it is more related to our traditions. This is different from Americans. They share everything. Everything is okay.”- Chinese-Cantonese-speaking FG Participant |
|  | Healthcare Seeking Involves Navigating Western and Traditional Medicine Due to Culturally Embedded Social Norms. | “People used to say it was the mammal called bat that causes it and that it can be healed with traditional medicine.” - Ethiopian FG Participant  “As someone who is born and raised most of my life in Africa, I mean, even people who are still here in the US with African descent, most people still seek both Western medicine and then they seek traditional medicine. So, at least everybody has some medicine, traditional medicine from home that they are hopping on to at home. But at the same time, they also seek Western medicine.” - Ghanaian Key Informant Interviewee  “They have mixed, like for using the traditional medicine and the Western medicine.” - Micronesian Key Informant Interviewees  “Sometimes, we don’t follow Western medication but Vietnamese medicinal herbs or other types of medication, some people may introduce good doctor, or they may know some useful information that can help us.” - Vietnamese FG Participant  “African people believe if they go get tested at the hospital for any disease the doctors will see more than what they expect. And they believe in herbs and think herbs will cure it.” - Nigerian FG Participant  “In terms of those that are still practicing shamanism… I think it's preventing our community from seeking treatment, and also continuing treatment, not just for liver cancer and hepatitis, but other medical conditions, because without the continuation of treatment, you're going to allow the disease to further progress.” – Hmong FG Participant  “When you have a conversation with the doctors, the word gets out on the street. So, a lot of people stay away from actually getting a diagnosis, proper diagnosis, timely diagnosis, so they end up going to the herbal doctors where privacy is a little bit assured.” - Nigerian and Ghanian FG Participant |
